# Nurse Marion's Romance

**Published:** 1887-04-30

**Authors:** Kate Furley


### Nurse Marion's Romance.

By Kate Furley.

### Chapter VI.—The Widow Explains.

It was an utterly new sensation to Nurse Marion to feel the wretchedness that beset her that evening. She had known loss and sorrow before, but never this consciousness of being out of tune with life. Everything was wrong: she could not interest herself in her work as she had been used to do; and yet the strong impulse which had hitherto drawn her to Jack Holtayne's side had suddenly failed. She said to herself that she would not be moved by Dr. Brandreth's words, would not change her conduct in any point to please his scruples, prejudices, or jealousies; but it surprised her to find that she was much more inclined to obey his wishes than to defy them. She began to analyse the effect Holtayne's presence had had upon her during the last fortnight, and the self-examination was far from satisfactory. She had been proud of the honour and love in which she had been held at St. Lazarus', and it vexed her to feel that she had declined in however slight a degree from her high altitude.

To counterbalance this she had gained—what? A fretful restlessness, an insatiate longing, a heartache, and now a miserable, indefinite jealousy for which she despised herself. She had offended and thrown away a loyal friend, and had thereby hardly gained a lover whose impatience and caprice were his most marked characteristics. Everyone seemed an enemy to-night—Dr. Brandreth for hinting at a fault in her conduct, Holtayne for placing her in a position that rendered reproof possible, and herself most of all for not having had strength to go her way unmoved by the opposing influences around her. She told herself that she loved Jack, that she was sure Jack loved her, and that she would not debar herself of a moment of his society to please the Doctor; but while she repeated her determination to herself, she continued to stay by herself in the garden; and she was angrily conscious that if the alternative were forced upon her she would rather lose Jack Holtayne's love than Dr. Brandreth's respect.

It was with an unwillingness that bewildered and annoyed her that she looked in on Jack before she retired for the night, and the greeting she received from her patient was not such as to compensate her for what she had endured that day on his behalf.

"Well, are you to be turned out?" he asked, abruptly.

"No; Dr. Brandreth has been more gentle with me than I deserved."

"I am glad of that; I suppose he concluded he would lose more than he'd gain by it; but he is a confounded prig. Still, as there's no fuss to be made about to-day, I think you might have come and spent the evening with me. It's very dull when you're not here to talk to me; and as I probably shall not be a trouble to you much longer, I think you might spare me an hour or two for the sake of old days."

"You are tired to-night," was all she said, and smoothed his tumbled pillow. As she did so the letter of the afternoon slipped from under it, and fell on the floor.

"What shall I do with this?" she asked.

"Oh! burn it! No, don't; that would be regarded as a crime on my part, and one is always supposed to keep a lady's letters—while it is likely to be found out that one has destroyed them. Put it under my pillow again."

Decidedly, thought Marion, Jack was out of temper that evening; and she went away with a sigh. She assured herself that it was weakness that made him so fretful, and that perhaps he had been worrying himself with the thought that he had brought some annoyance upon her; but her feminine instinct made her feel sure that the real cause of his irritation was the lady of the swallow-sealed letter; and she felt so angry with everybody for making rough her hitherto smooth path, and with herself for being angered thereat, that she ended by wishing that Dr. Brandreth, and Jack Holtayne, and the lady of the letter were all a hundred fathoms deep in the sea—especially the unknown lady.

The next morning found her restored to herself, and better able to go through the work she had to do. She could think over the previous day with more calmness, and resolve to show all whom it might concern that it was within her power still to behave with dignity and self-respect.

She had given Jack his breakfast, and was about to leave him, when he said, in a voice that was meant to simulate indifference: "By-the-bye, I suppose I can see visitors now if anyone takes the trouble to call on me. I feel well enough for it."

"I see no reason why you should not," she answered quietly, though her mind at once recalled the letter, with a slight return of inquisitive jealousy; "but you must ask the Doctor about it."

In the afternoon the visitor arrived. "The Swallow," Marion had mentally dubbed her in advance; and her costume carried out the colouring of the bird, if her expression and bearing suggested something more flaunting and less agile than the messenger of spring. It was the black and white of modified widowhood, so arranged as to convey only a vague reminiscence of bereavement, overlaid with possibilities of consolation. The lady was younger than herself, Marion thought; but there was a maturity of expression in her handsome Eurasian features, which made the nurse seem the junior. So at least it appeared to Jack Holtayne, as he saw them together for a moment when Marion ushered the visitor into the room. The olive-skinned lady with the dark flashing eyes seemed vulgar, handsome as she was, compared with the nurse, whose delicate features had gained only a deeper beauty through years passed in unselfish work.

He felt inclined to sigh; but instead he composed his features to a look of deepest tenderness, and murmured, as the door closed upon Marion,—"Mrs. Durrant—Clara—how kind this is of you!"

Mrs. Durrant remained with Holtayne for nearly an hour, and requested a few words with the nurse in private before she left.

There seemed an intuitive hostility between the two women, though Marion courteously asked the stranger to come into the farm-house parlour, and offered her a chair.

"I suppose there will be no objection to my coming to see Mr. Holtayne while he is here," said Mrs. Durrant.

"None, if the Doctor does not find the excitement of your visits injurious to him."

"Oh! there's no fear of that; seeing *me* won't hurt him," said the lady in a marked tone, playing with a bangle on her wrist. "I—I suppose he'll get quite strong again?"

"I hope so."

"I mean, there's nothing constitutional in his ailment—no important organs affected?"

"None; all that he requires is rest, quiet, and care."

"That's a good thing!" And Mrs. Durrant looked greatly relieved. "I should like to have him removed to my hotel. Can it be done?"

"Dr. Brandreth must be consulted about that. He will not permit any patient of his to be moved until it is quite safe for him to go."

"Oh! I would take good care of him—look after him quite as well as you can here, I have no doubt! Indeed, I wouldn't mind engaging you to nurse him till he is quite well—though you are not at all what I expected a nurse to be—you're far too good-looking."

"Ugliness is no longer considered an essential qualification for our work," said Marion with a slight smile; "but I don't undertake any but hospital work, except when specially requested by the matron."

"No doubt I can get somebody equally well qualified," answered Mrs. Durrant lightly. When Marion had expressed her acquiescence in this statement, she favoured her with a few details of personal history; for she seemed of a garrulous, good-tempered nature, that must needs talk to somebody.

"It was very vexatious that Mr. Holtayne should have had to come home when he did. The doctors insisted on his being shipped off at once—and indeed he was so awfully weak that I was afraid to protest. I couldn't come at the same time, for my little girl was laid up with an attack of fever, and I was forced to stay with her. She isn't such a very little girl either. You wouldn't think I had a daughter nearly ten years old, would you?"

"No."

"You see, I was married before I was seventeen. Mr. Durrant fell in love with me on the voyage to India, when I was going home from school, and he would take no refusal. He was much older than I, and had such a temper! Ah, well! he's dead now, poor man; but my Clara is his child all over; and a pretty time I had when she was getting better, and I so anxious about poor Mr. Holtayne, too! But I've brought her home, and mean to send her to a boarding-school, and then she'll find out by contrast what an indulgent mother I have been."

She rose and moved towards the door; but when Marion thought she was about to leave the room, she paused, and a faint touch of embarrassment appeared on her face, as she produced her purse.

"Mr. Holtayne tells me you have been very kind to him," she said. "Of course I'll give a handsome donation to the hospital; but will you take this to buy yourself a ribbon or something?" As she spoke, she took out some money, glancing with pity at the nurse's dress, the conventual simplicity of which struck her as being inevitably connected with poverty.

Marion's cheeks flushed, but a single look at Mrs. Durrant's face convinced her that only kindness was meant. "I thank you, but we do not accept gifts from patients or their friends," she answered; "and as we are all compelled to wear this uniform, ribbons are not of much use to me."

"Compelled to wear that dowdy dress—though I must admit it suits you wonderfully! Well, that is hard! I am sorry if I offended you; I meant no harm. And I shall be back to-morrow." And so Mrs. Durrant took herself off.

(*To be concluded.*)

